# Bicondylar Hoffa Fracture Successfully Treated with Headless Compression Screws

**DOI:** 10.1155/2014/139897

**Published:** 2014-07-16

**Authors:** Sang Yang Lee, Takahiro Niikura, Takashi Iwakura, Yoshitada Sakai, Ryosuke Kuroda, Masahiro Kurosaka

**Affiliations:** Department of Orthopaedic Surgery, Kobe University Graduate School of Medicine, 7-5-1 Kusunoki-cho, Chuo-ku, Kobe 650-0017, Japan

## Abstract

Bicondylar coronal plane fracture, eponymically named Hoffa fractures, is an extremely rare injury. We present a case of isolated unilateral bicondylar Hoffa fracture that was successfully treated with open reduction and internal fixation using headless compression screws with satisfactory results. We inserted posteroanteriorly oriented Acutrak screws perpendicular to the fracture plane via lateral parapatellar arthrotomy, which provided excellent compression across the fracture.

## 1. Introduction

Isolated coronal plane fractures of the distal femoral condyle, originally described by Hoffa [1], are rare, representing only 0.65% of all femoral fractures [2]. The eponymically named Hoffa fracture usually involves a single femoral condyle, most commonly the lateral femoral condyle [2, 3]. Bicondylar involvement is extremely rare, and, to the best of our knowledge, only 11 cases have been reported thus far in the English literature [4–13]. We describe a case of bicondylar Hoffa fracture that was successfully treated with headless compression screws.

## 2. Case Presentation

**Figure 1:**
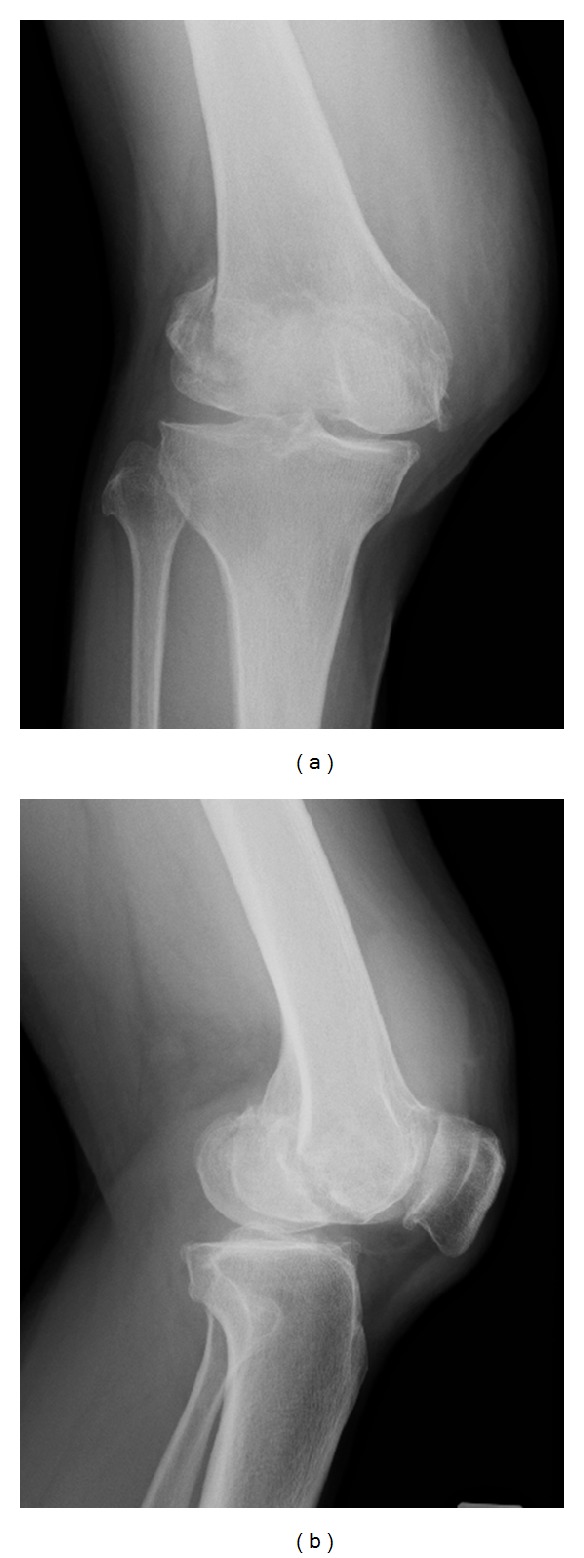
Initial posttraumatic anteroposterior (a) and lateral (b) radiographs of the right knee.

**Figure 2:**
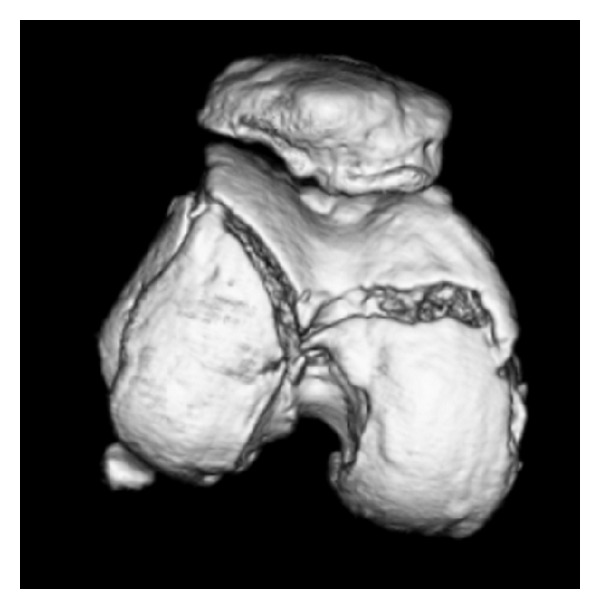
A three-dimensional computed tomographic image of the femoral articular cartilage of the right knee, showing the bicondylar Hoffa fracture.

A 63-year-old woman was admitted to the emergency department after being hit by a car while walking. Plain radiography (Figure 1) and computed tomography (Figure 2) revealed an isolated bicondylar Hoffa fracture on the right knee. The lateral and medial Hoffa fragments were dislocated. Eleven days after the accident, open reduction and internal fixation were performed. Under general anesthesia, the patient was placed in the supine position with the right limb exsanguinated.

**Figure 3:**
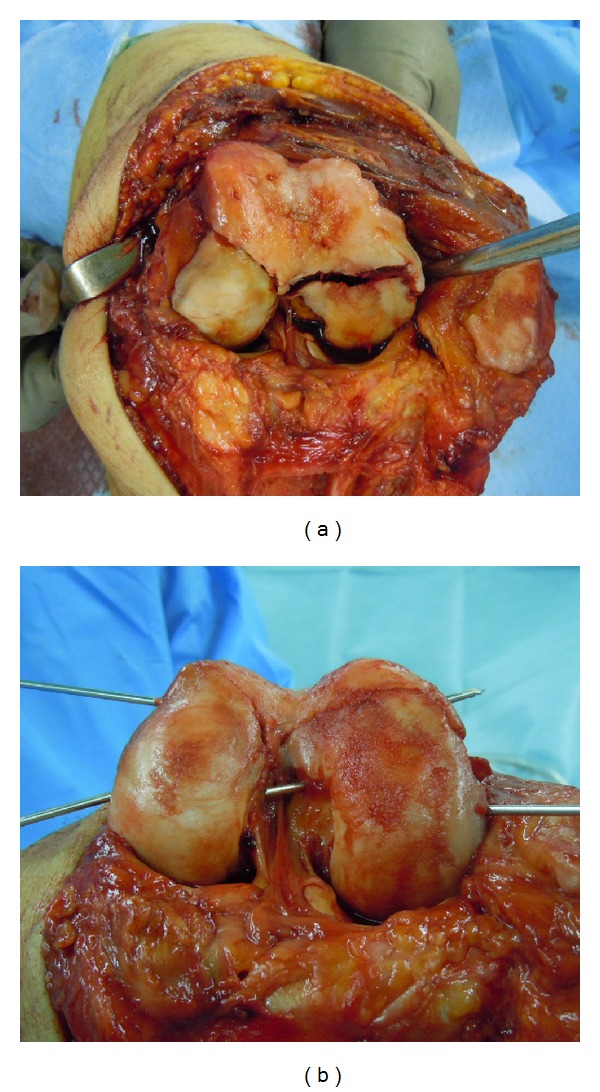
Intraoperative photographs illustrating the exposure of the bicondylar Hoffa fracture through a lateral parapatellar arthrotomy (a) and provision reduction with Kirschner wires (b).

**Figure 4:**
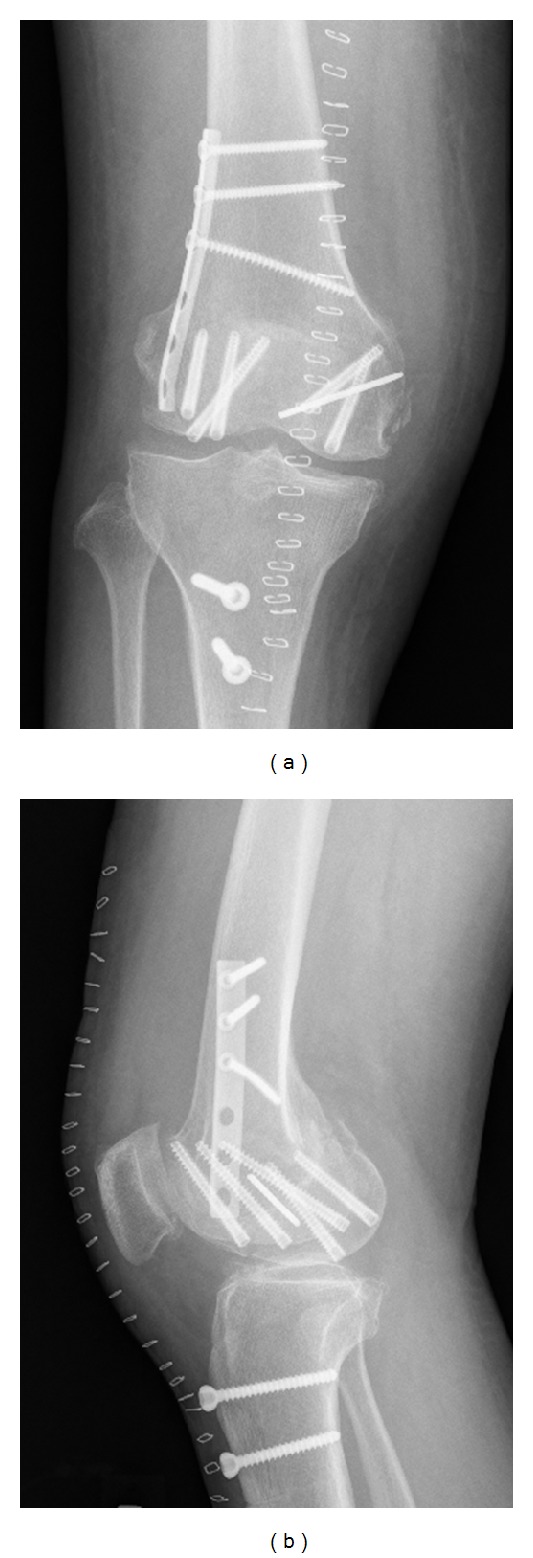
Anteroposterior (a) and lateral (b) radiographs obtained right after surgery.

Lateral parapatellar arthrotomy was performed. Tibial tubercle osteotomy was required to expose the intra-articular fragments. This revealed displaced Hoffa fractures of both femoral condyles (Figure 3(a)). Degenerative changes in the medial femoral cartilage were observed. The knee was flexed to allow initial, manual anterior delivery of the Hoffa fragments. The fragments were, then, anatomically reduced and 2 mm multiple Kirschner wires were inserted for temporal reduction and stabilization (Figure 3(b)). Subsequently, the fragments were compressed with a large, pointed reduction clamp. Anatomical reduction was confirmed by fluoroscopy and direct visualization of the articular surface. Six (three per fragment) Acutrak 4/5 headless compression screws (Acumed, Hillsboro, OR, USA) were inserted over the 1.4 mm Kirschner wires in the deep flexion position at the posterior articular surface directing anteriorly perpendicular to the fracture plane to compress the fractures (Figure 4). These screws were sunk to just below the cartilage bone interface. Finally, the fractures of the lateral wall of the lateral condyle were fixed with a 3.5 mm 1/3 tubular plate (Synthes, Oberdorf, Switzerland). The tubercle osteotomy was repaired using two 4.5 mm cortical screws (Synthes).

**Figure 5:**
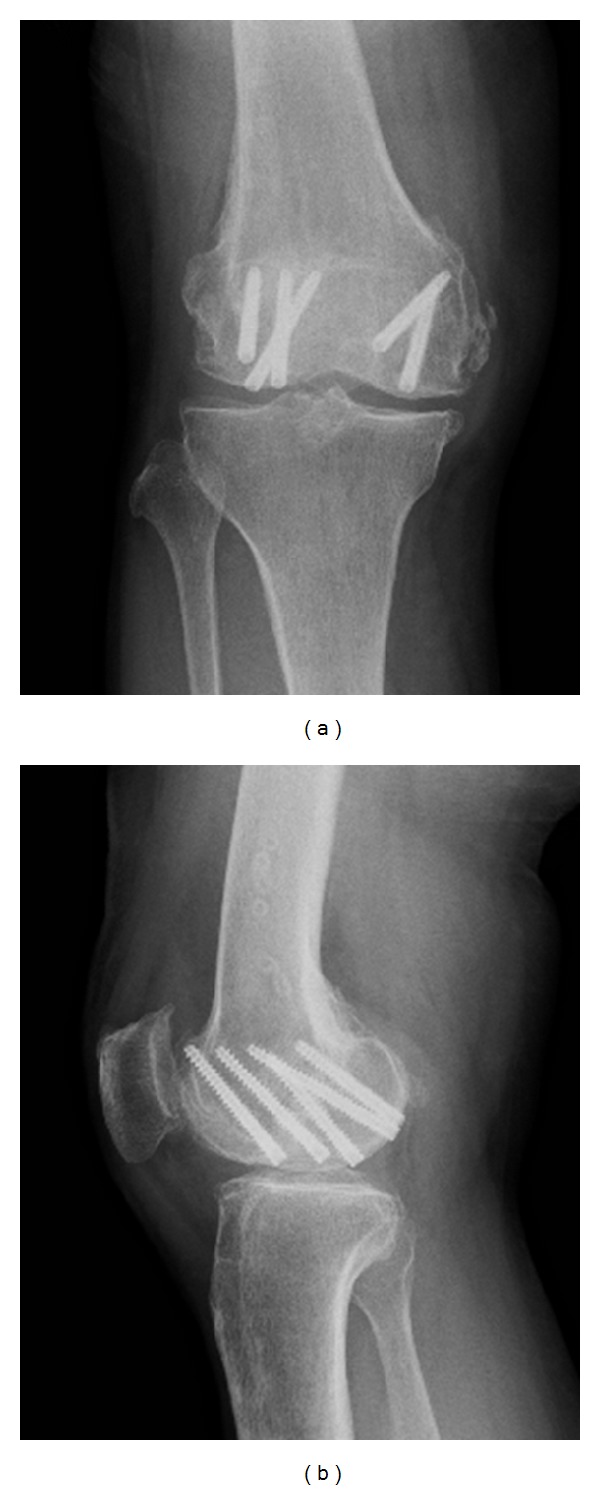
Anteroposterior (a) and lateral (b) radiographs obtained at the latest follow-up, at 2 years and 7 months after surgery.

Four days after surgery, intermittent knee mobilization was started along with isometric muscle strengthening exercise. Partial weight-bearing was permitted at 6 postoperative weeks and full weight-bearing at 10 postoperative weeks. Six months after the operation, radiography revealed fracture union, and the tubular plate and 3.5 mm/4.5 mm screws were removed. At the latest follow-up, at 2 years and 7 months after surgery, the patient was able to walk without discomfort and could perform all her daily and working activities normally. A postoperative radiological progression of osteoarthritis was not evident (Figure 5). The range of motion of the knee was 10–115°.

## 3. Discussion

Hoffa fracture (AO/OTA classification: 33-B3.2 [14]) generally results from severe high-energy trauma secondary to motor vehicle accidents or a fall from a height [2–4]. The specific mechanism of the injury that produces Hoffa fractures remains unknown. Lewis et al. [15] suggested that axial load to the femoral condyle when the knee is flexed to >90° produces posterior tangential fractures. Since the lateral femoral condyle has a greater anteroposterior (AP) dimension and the knee has a physiologically valgus orientation, the lateral condyle is more commonly involved. In our case, the bicondylar fracture might have been caused by a posterior and upward directed force with a hyperflexed knee without any varus or valgus, as suggested by Ul Haq et al. [13].

Conservative treatment of displaced Hoffa fracture with plaster cast was reported to lead to nonunion [8] or deformity, joint contracture, and subsequent osteoarthritis [15, 16]. Most authors recommend open reduction to restore normal condylar anatomy and rigid internal fixation, allowing functional recovery [4–13, 15, 16]. Lag screw fixation is the most accepted method to fix Hoffa fractures. A midline incision with medial/lateral parapatellar arthrotomy is the most commonly reported approach [10, 13, 17, 18]. Lateral parapatellar arthrotomy provides visualization of fractures and articular surface that is necessary for achieving perfect anatomical reduction and the exposure required to compress and rigidly fix the fractures with multiple lag screws [17, 18].

No consensus has been reached on the fixation method in terms of the anterior/posterior direction of screw insertion and type/number of screws to use. Although Hoffa fractures are typically fixed with AP-oriented screws [8–13], Jarit et al. [19] showed that fixation with posteroanteriorly- (PA-) oriented lag screws was biomechanically superior to AP-oriented lag screws when subjected to vertical loads. However, PA screw fixation requires the recession of the screw heads beneath the articular surface, which creates a large cartilage defect (i.e., >8.0 mm for a 6.5 mm cancellous screw). Headless compression screws can reduce the degree of required cartilage damage [20]. In our case, we used six (three per fragment) Acutrak headless compression screws in the deep flexion position at the posterior articular surface directing anteriorly perpendicular to the fracture plane (Figure 4). The screws we used were conical, with a minimal diameter of 4 mm and a maximum diameter of 5 mm. This is the first report describing the use of such screws for the treatment of a bicondylar Hoffa fracture. One advantage of using these screws is that compression along the entire length of the screw can be achieved, possibly resulting in improved stability compared with a conventional lag screw [21].

In conclusion, we describe a rare case of a bicondylar Hoffa fracture treated successfully with open reduction and internal fixation using Acutrak headless compression screws. We inserted six (three per fragment) PA screws perpendicular to the fracture plane via lateral parapatellar arthrotomy and achieved excellent compression across the fracture.
